# Magnitude of Additional Meal Consumption and Associated Factors Among Pregnant Women Attending Antenatal Care at Aleta Wondo Primary Hospital, Sidama Region, Ethiopia

**DOI:** 10.1155/tswj/9232431

**Published:** 2026-09-08

**Authors:** Amelo Bolka, Temesgen Tesfaye

**Affiliations:** ^1^ School of Public Health, Yirgalem Hospital Medical College, Yirgalem, Ethiopia; ^2^ Aleta Wondo Town Administration, Aleta Wondo Primary Hospital, Aleta Wondo, Ethiopia

**Keywords:** additional meal, antenatal care, associated factors, pregnant women, Sidama Region

## Abstract

**Background:**

Pregnancy increases nutrient requirements, making additional meal consumption essential for maternal and fetal health. However, although maternal nutrition studies in Ethiopia have largely focused on dietary diversity, evidence on additional meal consumption remains limited. This study is aimed at determining the magnitude of additional meal consumption and its associated factors among pregnant women receiving antenatal care (ANC) at Aleta Wondo Primary Hospital in the Sidama Region, Ethiopia.

**Method:**

A facility‐based cross‐sectional study was conducted among 407 pregnant women from December 2023 to February 2024. Participants were selected using systematic random sampling. Data were collected using a structured, pretested questionnaire. Additional meal consumption was defined as consuming at least one extra meal per day beyond the regular three meals (breakfast, lunch, and dinner). Factors associated with additional meal consumption were analyzed using logistic regression models, and the results were presented as adjusted odds ratios (AORs) with corresponding 95% confidence intervals (CIs).

**Result:**

The magnitude of additional meal consumption among pregnant women was 60.4% (95% CI: 55.6%–65.1%). Lower odds of consumption were observed among women with no formal education (AOR = 0.26, 95% CI: 0.10–0.66), those earning ≤ 3000 Ethiopian Birr (AOR = 0.28, 95% CI: 0.13–0.61), and those earning 3001–6000 Birr (AOR = 0.38, 95% CI: 0.17–0.86), corresponding to 74%, 72%, and 62% reductions, respectively, compared with their counterparts. Higher odds were observed among women without fear of weight gain (AOR = 3.55, 95% CI: 1.81–6.96), those with planned pregnancies (AOR = 3.23, 95% CI: 1.67–6.25), urban residents (AOR = 2.22, 95% CI: 1.27–3.88), and those from food‐secure households (AOR = 1.86, 95% CI: 1.11–3.12).

**Conclusion:**

The magnitude of additional meal consumption was 60.4%. The factors associated with additional meal consumption included educational status, place of residence, pregnancy planning, fear of weight gain, monthly household income, and household food security status. Integrating maternal nutrition into poverty alleviation and food security programs, as well as promoting community awareness of optimal dietary practices, is recommended.

## 1. Background

Adequate nutrition during pregnancy is essential for maintaining maternal health and supporting optimal fetal growth and development [1]. During this period, women experience increased energy and nutrient requirements due to physiological changes, placental development, and fetal growth [2]. To meet these increased nutritional demands, pregnant women are recommended to consume at least one additional meal beyond their usual daily meals, particularly during the second and third trimesters when energy and nutrient requirements are greatest [3].

The failure to take extra meals during pregnancy might lead to insufficient nutritional consumption and maternal malnutrition [2]. Maternal malnutrition during pregnancy can lead to complications such as increased susceptibility to infections, anemia, weakened immune function, fatigue, reduced productivity, obstructed labor, and higher maternal mortality for the mother. On the part of infants, it can result in increased fetal and neonatal mortality, intrauterine growth restriction, low birth weight, preterm birth, impaired immune function, birth defects, cretinism, and lower cognitive abilities [4–6]. It further negatively impacts the socioeconomic conditions of families and communities [7].

In industrialized nations, expectant mothers follow the more scientific and evidence‐based dietary recommendations of three meals plus one or more additional snacks. A study conducted in the United States revealed that over three‐quarters of participants followed this dietary practice [8]. Conversely, in many African developing nations, maternal nutrition remains insufficient to meet the increased dietary needs of pregnancy. This inadequacy contributes to poor nutritional status, diet‐related illnesses, and adverse reproductive health consequences [7].

In developing nations such as Ethiopia, adequate dietary habits and additional meal intake among pregnant women remain alarmingly insufficient [9, 10]. A systematic review and meta‐analysis in Ethiopia found that about 65.7% of pregnant women had poor dietary practices, and 80% consumed only three or fewer meals per day [9]. Moreover, a study in southern Ethiopia highlighted that additional meal practices and meal frequency among pregnant women were inadequate to meet their heightened energy and nutrient needs. Only 34.8% of pregnant women consumed extra meals or snacks between main meals, whereas 67.8% ate once or twice daily, and just 25.6% ate three or four times daily [11].

Several determinants of additional meal consumption during pregnancy exist in Ethiopia. These include sociodemographic and maternal characteristics among others [12]. Studies carried out in various areas of Ethiopia have found that maternal education, household income level, food insecurity level, place of residence, whether pregnancy is intended, nutrition knowledge, source of nutrition information, and attitudes toward gaining weight during pregnancy are among the major determinants of additional meal consumption [13–15]. All of these characteristics can affect a woman′s ability and willingness to satisfy her increased nutritional demands during pregnancy [16].

Various programs and strategies have been introduced to enhance maternal nutrition. Additionally, substantial progress in women′s nutritional status is essential to meet Sustainable Development Goal (SDG) 2, ending hunger, and SDG 3, focusing on health. The Ethiopian government developed the National Nutrition Programme II (NNPII), an intervention strategy centered on the first 1000 days of life, aiming to combat undernutrition [17]. Despite these efforts, the prevalence of adequate dietary practices, including additional meal intake, among pregnant women in Ethiopia remains insufficient [9].

Although evidence exists on overall dietary practices and related factors among pregnant women in Ethiopia, there is a lack of information specifically addressing additional meal or snack consumption during pregnancy. This study is aimed at addressing this gap by contributing valuable insights. Accordingly, the research focuses on evaluating additional meal practices and identifying associated factors among pregnant women in Aleta Wondo town, Sidama Region, Southern Ethiopia.

## 2. Methods and Materials

### 2.1. Study Setting, Design, and Period

A facility‐based cross‐sectional study was conducted from December 20, 2023, to February 15, 2024, at Aleta Wondo Primary Hospital in Aleta Wondo town, located in the Sidama Region of Southern Ethiopia. Aleta Wondo town is situated 64 km from Hawassa, the regional capital, and 339 km south of Addis Ababa, Ethiopia′s capital. The town is surrounded by the Aleta Wondo district administration. The local inhabitants primarily depend on subsistence farming, with key crops including maize, root crops such as Ensete (false banana), haricot beans, and cash crops like coffee and khat. In 2024, the hospital provided antenatal care services to 9700 pregnant women.

### 2.2. Study Population and Their Eligibility Criteria

All pregnant women who received antenatal care at Aleta Wondo Primary Hospital in 2023–2024 constituted the source population. The study population included those who attended the hospital during data collection, excluding women who were critically ill or unable to communicate.

### 2.3. Sample Size Determination

The sample size was determined using the single population proportion formula, based on a 95% confidence level, a 5% margin of error, and an assumed proportion of 50%, given the absence of previous studies in a comparable context. A 10% allowance was added to account for potential nonresponse, resulting in an initial sample size of 422. Since the source population was finite (*N* = 9700), the sample size was further adjusted using the finite population correction formula, and the final sample size was determined to be 407.

### 2.4. Sampling Technique

This study used systematic sampling to recruit participants. The sampling frame was based on ANC service records. During the 2 months before data collection, an average of 778 women received ANC services. The sampling interval was calculated by dividing the total number of ANC clients by the required sample size (*k* = *N*/*n* = 778/407 = 1.91 ≈ 2). Participants were then selected by choosing every second woman, with the first participant randomly chosen by lottery from the first two women.

### 2.5. Study Variables

The outcome variable of interest was additional meal consumption. On the other hand, 14 independent variables were considered potential confounders in the study (variables with a *p* value < 0.25 in the bivariable analysis). These were as follows: [1] women′s age, [2] pregnant women education status, [3] husband′s education status, [4] parity, [5] gravidity, [6] family size, [7] place of residence, [8] monthly income level, [7] food security (secured, insecure), [9] pregnancy plan, [10] antenatal care visit frequency, [11] knowledge, [12] attitude, and [13] fear of weight gain.

Pregnant women who consumed at least one additional meal per day, in addition to their regular meals (breakfast, lunch, and dinner), were considered to have consumed an additional meal [18, 19].

Pregnant women who consumed at least five different food categories were classified as having a high dietary diversity score. This score was determined by tallying the number of unique food groups consumed over a 24‐h recall period. A score of less than five signified a lower degree of dietary diversity [20].

Pregnant women′s nutritional knowledge on dietary practices was assessed with 10 questions, whereas their attitudes toward these practices were measured using nine questions. Each response was assigned a score: correct answers received one point, whereas incorrect answers received zero. Internal consistency reliability of the knowledge and attitude items was assessed using Cronbach’s alpha (*α* = 0.814). A total score was computed for both knowledge and attitude domains. Knowledge was classified as good if the score met or exceeded the mean, and poor if it fell below the mean [11]. Likewise, attitudes were deemed positive if above the mean and negative if at or below the mean [21].

### 2.6. Data Collection Instruments and Procedures

The data were collected using structured, interviewer‐administered questionnaires that were developed following an extensive review of relevant literature [21–23]. The instrument comprised five sections: sociodemographic characteristics, obstetric and pregnancy‐related variables, knowledge and belief‐related questions, household environmental and food security factors, and items assessing additional meal practices and associated determinants.

Initially, the questionnaire was prepared in English, then translated into Sidamu Afoo (the local language), and subsequently back translated into English by a linguistic expert to ensure consistency and accuracy. The final data collection was carried out using the Sidamu Afoo version of the questionnaire.

Data collection was carried out by a team of four midwives and one supervisor through face‐to‐face interviews. The data collectors and supervisor received 2 days of training on the study objectives, significance of the research, participants′ rights, informed consent procedures, data collection tools, use of the KoboToolbox system, and effective interviewing techniques. Selected pregnant women were interviewed in a private setting after their routine service appointments.

Dietary diversity was assessed using a 24‐h recall method adapted from the Food and Agriculture Organization (FAO). Participants were asked to report all foods and beverages consumed in the previous 24 h, including those eaten both at home and outside. The Women′s Dietary Diversity Score (WDDS) was calculated by summing the number of food groups consumed across 10 categories: grains, white roots and tubers, and plantains; pulses (beans, peas, and lentils), nuts and seeds; milk and milk products; meat, poultry, and fish; eggs; dark green leafy vegetables; other vitamin A‐rich fruits and vegetables; other vegetables; and other vegetables [24].

Household food security was measured using the Household Food Insecurity Access Scale (HFIAS), which assesses nine occurrence‐based questions reflecting experiences of food insecurity over the previous 4 weeks. Based on the responses, households were categorized as either food secure or food insecure [25].

Nutritional status was assessed using mid‐upper arm circumference measured with a nonstretchable MUAC tape. The midpoint between the acromion and olecranon processes of the left arm was identified and marked, after which the tape was placed around the relaxed arm at the marked point and the measurement recorded to the nearest 0.1 cm [26–28]. Pregnant women with MUAC < 23 cm were classified as undernourished, whereas those with MUAC ≥ 23 cm were considered to have normal nutritional status [29].

### 2.7. Data Quality Assurance

Experienced data collectors and supervisors received comprehensive training on the use of the KoboToolbox platform for data collection. The data collection tool was pretested using 5% of the total sample at Aleta Chuko Primary Hospital, and necessary modifications were made based on the pretest findings. The use of KoboToolbox facilitated structured data entry and improved data accuracy. Supervisors reviewed completed questionnaires daily to ensure completeness, consistency, and reliability. The principal investigator closely supervised and coordinated the overall data collection process.

### 2.8. Data Analysis Methods

Data were collected using the KoboToolbox platform and subsequently exported to IBM SPSS Statistics 26 for coding, cleaning, and analysis. Descriptive statistics, including frequencies, measures of central tendency, and dispersion, were used to summarize the data.

Bivariable and multivariable logistic regression analyses were performed to identify factors associated with the outcome variable. Variables with a *p* value < 0.25 in the bivariable analysis were entered into the multivariable logistic regression model to control for potential confounders. Model fitness was assessed using the Hosmer–Lemeshow goodness‐of‐fit test (*p* = 0.894), indicating an adequate fit. Multicollinearity among independent variables was examined using the variance inflation factor (VIF ≤ 1.853). In the multivariable analysis, a *p* value < 0.05 was considered statistically significant. The strength of associations was reported using adjusted odds ratios with corresponding 95% confidence intervals.

## 3. Results

### 3.1. Sociodemographic Characteristics

A total of 399 pregnant women participated in the study, yielding a response rate of 98%. The mean age (±SD) of the participants was 26.8 ± 3.9 years, with the majority (79.4%) aged between 25 and 34 years. Nearly all respondents (98.5%) were married. Regarding ethnicity, 74.9% of participants were of Sidama origin, and 34.6% identified as followers of the Apostles′ Creed (a distinct group that adheres to a non‐Trinitarian theology and emphasizes baptism in the name of Jesus Christ alone). About half (51.8%) of the women and 60.2% of their husbands had attained secondary education or higher. In terms of occupation, 59.6% of the participants were housewives, whereas 61.7% of their husbands were engaged in merchant activities. Slightly less than two‐thirds (64.4%) of respondents resided in urban areas. The majority (82.2%) lived in households with fewer than five members. The median family size was 3, with an interquartile range (IQR) of 2. Two‐thirds (66.4%) of participants reported that the nearest health facility was within a 30‐min distance (Table 1).

**Table 1:** Sociodemographic and economic characteristics of pregnant women attending antenatal care at Aleta Wondo Primary Hospital, Sidama Region, Ethiopia.

Variables (*n* = 399)	Frequency	Percentage (%)
Age (years)		
< 25	53	13.3
25–34	317	79.4
> 35	29	7.3
Marital status		
Married	393	98.5
Others	6	1.5
Ethnicity		
Sidama	299	74.9
Amhara	60	15.1
Gurage	32	8
Others	8	2
Religion		
Protestant	173	43.4
Apostles′ Creed followers	138	34.6
Orthodox	72	18
Muslim	16	4
Study participants′ education status		
No formal education	53	13.3
Primary (1–8)	139	34.8
Secondary and above	207	51.8
Husband education		
No formal education	28	7.0
Primary (1–8)	131	32.8
Secondary and above	240	60.2
Study participants′ occupation		
Merchant	79	19.8
Employee	76	20.5
Housewife	238	59.7
Husband occupation		
Farmer	48	12.0
Merchant	246	61.7
Employee	105	26.3
Family size		
< 5 members	328	82.2
≥ 5 members	71	17.8
Place of residence		
Urban	257	64.4
Rural	142	35.6
Distance from nearest health facility		
≤ 30 min	265	66.4
> 31 min	134	33.6

### 3.2. Obstetrics Related Factors

Slightly less than two‐thirds (63.7%) of participants were multigravida. Four‐fifths (81.5%) had a parity of two or fewer prior births. The mean gestational age (±SD) was 29.6 ± 6.6 weeks. Most pregnancies (80.2%) were planned. Concerning ANC, 94.2% of respondents had attended at least one ANC visit, and 33.6% had completed two visits for the current pregnancy. Regarding interpregnancy interval, 62.5% (173/277) reported an interval greater than 24 months. The majority (83.7%) of participants did not report any illness in the month preceding the survey. Only 4.0% had a chronic illness, of whom 56.2% were diagnosed with hypertension (Table 2).

**Table 2:** Obstetric characteristics of pregnant women attending antenatal care at Aleta Wondo Primary Hospital, Sidama Region, Ethiopia.

Variable	Frequency	Percent (%)
Gravidity (*n* = 399)		
Primigravida	122	30.6
Multigravida (2–4)	254	63.7
Grand multigravida (≥ 5)	23	5.8
Parity (*n* = 399)		
≤ 2	325	81.5
≥ 3	74	18.5
Gestational age (*n* = 399)		
Second trimester	182	45.6
Third trimester	217	54.4
Pregnancy plan (*n* = 399)		
Yes	320	80.2
No	79	19.8
Previous ANC follow‐up (*n* = 277)		
Yes	261	94.2
No	16	5.8
Number of current ANC visits (*n* = 399)		
One visit	81	20.3
Two visits	134	33.6
Three visits	110	27.6
Four or more visits	74	18.5
Interpregnancy interval (*n* = 277)		
≤ 24 months	104	37.5
> 24 months	173	62.5
Illness in this month (*n* = 399)		
Yes	65	16.3
No	334	83.7
Chronic illness (*n* = 399)		
Yes	16	4.0
No	383	96.0
Type of chronic illness (*n* = 16)		
Hypertension	9	56.2
Asthma	2	12.5
Diabetes mellitus	3	18.8
Heart disease	2	12.6

### 3.3. Knowledge and Attitude Related Factors

Most participants (90.2%) received nutrition‐related information during pregnancy. The primary source of information was healthcare providers (64.4%), followed by media sources (18.9%). The mean knowledge score (±SD) was 7.5 ± 1.4, with 61.4% of respondents demonstrating good knowledge of maternal nutrition. The mean attitude score (±SD) was 23.2 ± 3.8, and 55.9% of participants exhibited a favorable attitude toward nutrition during pregnancy. Seventy‐two participants (18.0%) expressed concern about weight gain during pregnancy. Although 79.9% did not avoid any foods, 20.1% reported avoiding certain food items, primarily due to cultural beliefs (56.3%). Among those who practiced food avoidance, fruits were the most commonly avoided items (78.8%) (Table 3).

**Table 3:** Knowledge and belief characteristics of pregnant women attending antenatal care at Aleta Wondo Primary Hospital, Sidama Region, Ethiopia.

Variable	Frequency	Percent (%)
Nutrition information during pregnancy		
Yes	360	90.2
No	39	9.8
Source of information (*n* = 360)		
Health professionals	232	64.4
Family/relatives	47	13.1
Friends	13	3.6
Media	68	18.9
Knowledge		
Good	245	61.4
Poor	154	38.6
Attitude		
Positive	223	55.9
Negative	176	44.1
Fear of weight gain during pregnancy		
Yes	72	18.0
No	327	82.0
Avoided foods during pregnancy		
Yes	80	20.1
No	319	79.9
Reason for avoidance (*n* = 80)		
Culture	45	56.3
Aversion	28	35.0
Health related	7	8.7
Types of foods avoided (*n* = 80)		
White roots and tubers	5	6.2
Green leafy vegetables	5	6.2
Fruits	63	78.8
Animal products	7	8.8

### 3.4. Household Income and Food Security Related Factors

The median monthly household income was 4000 Ethiopian Birr (70.95 $US) (IQR = 4500), with 41.9% of participants earning ≤ 3000 Ethiopian Birr (53.2 $US) per month. Ownership of household assets varied: 73.9% owned a radio or television, 15.0% owned a refrigerator, and 39.6% owned a stove. Three‐fourths (75.4%) of participants reported owning farmland. Additionally, 41.6% owned cattle, and 43.9% owned chickens. Regarding household food security, 61.2% of participants were classified as food secure (Table 4).

**Table 4:** Household wealth and food security status of pregnant women attending antenatal care at Aleta Wondo Primary Hospital, Sidama Region, Ethiopia.

Variable (*n* = 399)	Frequency	Percent (%)
Household income		
≤ 3000 Eth Birr (53.2 $US)	167	41.9
3001–6000 Eth Birr (53.22–106.42 $US)	146	36.6
≥ 6001 Eth Birr (106.43 $US)	86	21.6
Own radio/television		
Yes	295	73.9
No	104	26.1
Own refrigerator		
Yes	60	15.0
No	339	85.0
Own stove		
Yes	158	39.6
No	241	60.4
Own farm		
Yes	301	75.4
No	98	24.6
Own cattle		
Yes	166	41.6
No	233	58.4
Own chicken		
Yes	175	43.9
No	224	56.1
Household food security		
Food secure	244	61.2
Food insecure	155	38.8

Abbreviation: Eth Birr, Ethiopian Birr.

### 3.5. Dietary Diversity and Nutritional Status of the Study Participants

**Figure 1:**
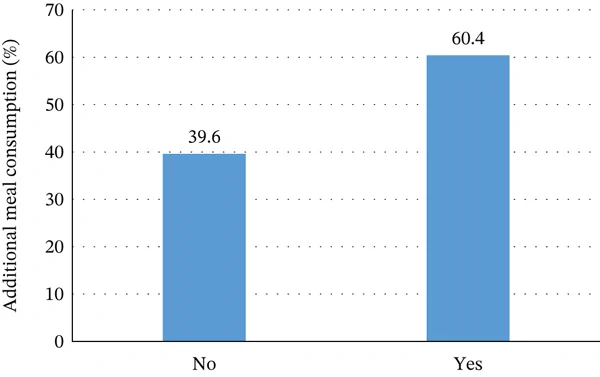
Magnitude of additional meal consumption among pregnant women attending antenatal care at Aleta Wondo Primary Hospital, Sidama Region, Ethiopia.

The mean dietary diversity score (±SD) among pregnant women was 4.7 ± 1.6. About half (51.4%) of participants achieved adequate dietary diversity. Cereals were the most commonly consumed food group (91.6%), followed by vegetables (80.4%) and tubers (62.4%). In contrast, consumption of animal‐source foods was relatively low: only 32.3% consumed milk and dairy products, and 29.8% consumed meat. The least consumed food group was vitamin A‐rich fruits, with only 12.7% of participants reporting intake (Figure 1). The mean (± SD) mid‐upper arm circumference of the pregnant women was 23.67 ± 1.93 cm. Overall, 12.8% (95% CI: 9.52%–16.08%) were undernourished (Table 5).

**Table 5:** Food groups consumed and nutritional status among pregnant women attending antenatal care at Aleta Wondo Primary Hospital, Sidama Region, Ethiopia.

Variable (*n* = 399)	Frequency	Percent (%)
Starchy staples		
Yes	365	91.6
No	34	8.4
Dark green leafy vegetables		
Yes	321	80.4
No	78	19.6
Other vitamin A‐rich fruits and vegetables		
Yes	243	60.9
No	156	39.1
Pulses (beans, peas, and lentils)		
Yes	219	54.9
No	180	45.1
Other vegetables		
Yes	209	52.4
No	190	47.6
Nuts and seeds		
Yes	153	38.3
No	246	61.7
Other fruits		
Yes	136	34.1
No	263	65.9
Milk and milk products		
Yes	129	32.3
No	270	67.7
Eggs		
Yes	79	19.8
No	320	80.2
Meat, poultry and fish		
Yes	51	12.8
No	348	87.2
Nutritional status		
Normal	348	87.2
Undernourished	51	12.8

### 3.6. The Magnitude of Additional Meal Consumption

The magnitude of additional meal consumption among pregnant women in this study was 60.4% (95% CI: 55.6%–65.1%). The mean meal frequency (±SD) was 3.6 ± 0.74 meals per day. Among participants who reported consuming additional meals, the majority (85.9%) had one extra meal, whereas a smaller proportion (14.1%) consumed two or more additional meals (Figure 1).

### 3.7. Factors Associated With Additional Meal Consumption

A total of fourteen variables were initially included in the bivariable logistic regression analysis. Of these, twelve variables with a *p* value < 0.25 were selected as candidates for the multivariable logistic regression model. In the final model, educational status, place of residence, pregnancy planning, fear of weight gain, monthly household income, and household food security status were found to be significantly associated with additional meal consumption (*p* < 0.05).

Pregnant women with no formal education were 74% less likely to consume additional meals compared with those with college‐level education or higher (AOR = 0.26, 95% CI: 0.10–0.66). Place of residence was also significantly associated with the outcome; urban residents had more than twice the odds of consuming additional meals compared with rural residents (AOR = 2.22, 95% CI: 1.27–3.88).

Pregnancy planning showed a strong positive association, as women with planned pregnancies were over three times more likely to consume additional meals compared with those with unplanned pregnancies (AOR = 3.23, 95% CI: 1.67–6.25). Fear of weight gain was another important factor; pregnant women who did not express concern about weight gain were 3.55 times more likely to consume additional meals than those who had such concerns (AOR = 3.55, 95% CI: 1.81–6.96).

Monthly household income demonstrated a significant gradient effect. Women from households earning ≤ 3000 Ethiopian Birr were 72% less likely (AOR = 0.28, 95% CI: 0.13–0.61), and those earning 3001–6000 Birr were 62% less likely (AOR = 0.38, 95% CI: 0.17–0.86), to consume additional meals compared with those with a monthly income ≥ 6001 Birr.

Finally, household food security status was significantly associated with additional meal consumption. Pregnant women from food‐secure households had 1.86 times higher odds of consuming additional meals compared with those from food‐insecure households (AOR = 1.86, 95% CI: 1.11–3.12) (Table 6).

**Table 6:** Factors associated with additional meal consumption among pregnant women attending antenatal care at Aleta Wondo Primary Hospital, Sidama Region, Ethiopia.

Variable (*n* = 399)	Additional meal		COR (95% CI)	AOR (95% CI)
	Yes	No		
Age				
≤ 24	32 (60.4)	21 (39.6)	2.49 (0.98–6.32)	0.82 (0.25–2.96)
25–34	198 (62.5)	119 (37.5)	2.72 (1.24–5.96)	1.21 (0.43–3.40)
≥ 35	11 (37.9)	18 (62.1)	1.00	1.00
Educational status				
No formal education	19 (35.8)	34 (64.2)	0.15 (0.07–0.32)	0.26 (0.10–0.66)^a^
Primary education	69 (49.6)	70 (50.4)	0.26 (0.14–0.49)	0.51 (0.24–1.10)
Secondary education	89 (70.6)	37 (29.4)	0.64 (0.33–1.23)	0.91 (0.42–1.95)
College or above	64 (79.0)	17 (21.0)	1.00	1.00
Residence				
Urban	183 (71.2)	74 (28.8)	3.58 (2.33–5.51)	2.22 (1.27–3.88)^a^
Rural	58 (40.8)	84 (59.2)	1.00	1.00
Family size				
< 5	212 (64.6)	116 (35.4)	2.65 1.57–4.47)	1.79 (0.84–3.82)
≥ 5	29 (40.8)	42 (59.2)	1.00	1.00
Gravidity				
Primigravida	88 (72.1)	34 (27.9)	5.92 (2.24–15.64)	2.14 (0.52–8.79)
Multigravida	146 (57.5)	108 (42.5)	3.09 (1.23–7.77)	1.73 (0.48–6.25)
Grand multigravida	7 (30.4)	16 (69.6)	1.00	1.00
Planned pregnancy				
Yes	215 (67.2)	105 (32.8)	4.17 (2.47–7.05)	3.23 (1.67–6.25)^a^
No	26 (32.9)	53 (67.1)	1.00	1.00
Number of ANC visits				
One visit	34 (42.0)	47 (58.0)	0.33 (0.17–0.63)	0.56 (0.25–1.26)
Two visits	84 (62.7)	50 (37.3)	0.76 (0.41–1.39)	1.63 (0.76–3.48)
Three visits	72 (65.5)	38 (34.5)	0.85 (0.46–1.60)	1.35 (0.64–2.89)
≥ 4 visits	51 (68.9)	23 (31.1)	1.00	1.00
Fear of weight gain				
No	32 (44.4)	40 (55.6)	0.45 (0.27–0.76)	3.55 (1.81–6.96)^a^
Yes	209 (63.9)	118 (36.1)	1.00	1.00
Knowledge				
Good	165 (67.3)	80 (32.7)	2.12 (1.40–3.20)	1.38 (0.80–2.37)
Poor	76 (49.4)	78 (50.6)	1.00	1.00
Attitude				
Positive	122 (54.7)	101 (45.3)	0.58 (0.38–0.87)	0.91 (0.54–1.54)
Negative	119 (67.6)	57 (32.4)	1.00	1.00
Household monthly income				
≤ 3000 Eth Birr	85 (50.9)	82 (49.1)	0.19 (0.09–0.36)	0.28 (0.13–0.61)^a^
3001–6000 Eth Birr	83 (56.8)	63 (43.2)	0.24 (0.12–0.46)	0.38 (0.17–0.86)^a^
≥ 6001 Eth Birr	73 (84.9)	13 (15.1)	1.00	1.00
Household food security status				
Secure	175 (71.7)	69 (28.3)	3.42 (2.24‐5.22)	1.86 (1.11–3.12)^a^
Insecure	66 (42.6)	89 (57.4)	1.00	1.00

Abbreviations: 1.00, reference; ANC, antenatal care; AOR, adjusted odds ratio; CI, confidence interval; COR, crude odds ratio; Eth Birr, Ethiopian Birr; n: sample size.

^a^Significant association.

## 4. Discussion

This study assessed the magnitude of additional meal consumption and its associated factors among pregnant women attending ANC services at Aleta Wondo Primary Hospital in the Sidama Region, Ethiopia. The findings indicated that 60.4% of pregnant women consumed additional meals during pregnancy. The prevalence of undernutrition among the pregnant women was 12.8%. Predictors of this practice were as follows: educational status, place of residence, pregnancy planning, fear of weight gain, monthly income, and household food security.

The present study revealed that 60.4% of pregnant women consumed extra meals or snacks between their main meals. This result aligns with findings from a study in Pakistan, where 57.3% of pregnant women reported consuming additional meals or snacks [30]. However, the magnitude observed in this study is higher than reports from some other studies in Nigeria (36.6%) [31], Gedeo Zone, southern Ethiopia (34.8%) [11], Wondo Genet district, southern Ethiopia (21.6%) [19], and Jille Tumuga district, northeast Ethiopia (44.7%) [32]. These variations may be attributed to differences in study settings, socioeconomic conditions, and levels of awareness regarding optimal maternal nutrition. The relatively higher prevalence observed in this study could be explained by the urban nature of the study area, where access to health services, nutrition counseling, and information dissemination is likely better.

Educational status was significantly associated with additional meal consumption. Pregnant women with no formal education were 74% less likely to consume additional meals compared with those with higher education. This finding is consistent with studies conducted in Southern Brazil [33], Dilla Zuria, Gedeo Zone [11], Illu Aba Bor Zone, southwest Ethiopia [23]. Education likely enhances awareness of proper dietary practices during pregnancy and is often linked with improved socioeconomic status, thereby increasing the ability to access adequate food.

Place of residence also showed a significant association, with urban residents having more than twice the likelihood of consuming additional meals compared with rural counterparts. Similar findings have been reported in Bale Zone of Ethiopia [34], Bench‐Sheko and Kaffa Zones of Ethiopia [21]. This may be due to better access to health services, improved exposure to nutrition‐related information, and greater food availability in urban settings. Additionally, lifestyle and economic differences between urban and rural populations may contribute to disparities in dietary practices.

Pregnancy planning was another important determinant. Women with planned pregnancies were more than three times as likely to consume additional meals compared with those with unplanned pregnancies. Although limited evidence exists in the Ethiopian context, previous studies suggest that planned pregnancies are associated with improved health behaviors, including better dietary practices [11, 35]. This may be because planned pregnancies are often accompanied by greater preparedness, increased healthcare utilization, and enhanced awareness of maternal nutritional needs [36].

Fear of weight gain was found to negatively influence additional meal consumption. Women who did not fear weight gain were significantly more likely to consume extra meals. This finding is supported by previous studies indicating that misconceptions about weight gain can reduce meal frequency during pregnancy [37]. In many sociocultural contexts, concerns about excessive weight gain may discourage adequate food intake, potentially compromising maternal and fetal health [38].

Household income demonstrated a strong association with additional meal consumption. Women from lower‐income households (≤ 3000 and 3001–6000 Ethiopian Birr/≤ 53.2 and 53.22–106.42 $US) were significantly less likely to consume additional meals compared with those from higher‐income households (≥ 6001 Birr/106.43 $US). This finding is consistent with studies conducted in Bahir Dar [39], Mizan Aman [22], Gondar [40], and Gedeo Zone [11]. Higher income levels enhance access to sufficient and diverse foods, whereas financial constraints may limit the ability to afford additional meals [41, 42].

Similarly, household food security status was a significant predictor. Pregnant women from food‐secure households were twice as likely to consume additional meals compared with those from food‐insecure households. This finding aligns with studies from Mizan Aman [22], West Gojam [43], and Illu Aba Bor [23]. Food security plays a crucial role in ensuring adequate dietary intake during pregnancy, whereas food insecurity can restrict access to sufficient and nutritious food, thereby negatively affecting maternal nutrition.

## 5. Limitation of the Study

This study has several limitations that should be considered when interpreting its findings. First, the assessment of dietary intake did not include detailed nutrient analysis, limiting insights into the quality and adequacy of nutrient consumption. Second, the reliance on self‐reported data may have introduced recall bias and social desirability bias, potentially affecting the accuracy of responses. Finally, although the questionnaire was adapted from previous studies and pretested, some constructs (particularly fear of weight gain and nutrition knowledge) were not assessed using internationally validated scales.

## 6. Conclusion

The study revealed that the magnitude of additional meal consumption among pregnant women was 60.4% in the study area. Key determinants of additional meal consumption included educational status, place of residence, pregnancy planning, fear of weight gain, monthly household income, and household food security status.

## 7. Recommendation

Aleta Wondo Primary Hospital, in collaboration with relevant stakeholders, should strengthen nutrition counseling during antenatal care services, with particular emphasis on the importance of additional meal consumption during pregnancy and addressing misconceptions about excessive weight gain. Interventions should particularly target women with lower educational attainment, those residing in rural areas, and women from food‐insecure or low‐income households.

**Nomenclature**
AOR: adjusted odds ratio
CI: confidence interval
CL: confidence level
DDS: dietary diversity score
FAO: Food and Agriculture Organization
HFIAS: Household Food Insecurity Access Scale
IQR: interquartile range
IRB: Institutional Review Board
SD: standard deviation
SDG: Sustainable Development Goal
WHO: World Health Organization

## Author Contributions

Study conceptualization and methodology: Temesgen Tesfaye and Amelo Bolka. Data curation, formal analysis, investigation, funding acquisition, and draft writing: Temesgen Tesfaye. Software, supervision, validation, and review and editing: Amelo Bolka.

## Funding

No funding was received for this manuscript.

## Ethics Statement

This study adhered to the Declaration of Helsinki. Ethical approval was granted by the Institutional Review Board (IRB) of Yirgalem Hospital Medical College (Protocol Number: YHMC/IRB061, Date: November 2, 2023). Written informed consent was obtained from the participants after a comprehensive explanation of the study′s purpose. Participant confidentiality was ensured by using coded identifiers to protect their data.

## Conflicts of Interest

The authors declare no conflicts of interest.

## Data Availability

The data that support the findings of this study are available from the corresponding author upon reasonable request.

## References

[1] World Health Organization , Nutrition Geneva, 2021, World Health Organization.

[2] WHO , WHO Recommendations on Antenatal Care for a Positive Pregnancy Experience, 2016, WHO. 28079998

[3] UNICEF , Maternal Nutrition: Preventing Malnutrition in Pregnant and Breastfeeding Women, 2020, UNICEF.

[4] Dumrongwongsiri O. , Winichagoon P. , Chongviriyaphan N. , Suthutvoravut U. , Grote V. , and Koletzko B. , Effect of Maternal Nutritional Status and Mode of Delivery on Zinc and Iron Stores at Birth, Nutrients. (2021) 13, no. 3, 10.3390/nu13030860, 33808021. PMC8001279 33808021

[5] Bhowmik B. , Siddique T. , Majumder A. , Mdala I. , Hossain I. A. , Hassan Z. , Jahan I. , Moreira N. C. V. , Alim A. , Basit A. , Hitman G. A. , Khan A. K. A. , and Hussain A. , Maternal BMI and Nutritional Status in Early Pregnancy and Its Impact on Neonatal Outcomes at Birth in Bangladesh, BMC Pregnancy and Childbirth. (2019) 19, no. 1, 1–14, 10.1186/s12884-019-2571-5. 31711436 PMC6849244

[6] Castrogiovanni P. and Imbesi R. , The Role of Malnutrition During Pregnancy and Its Effects on Brain and Skeletal Muscle Postnatal Development, Journal of Functional Morphology and Kinesiology. (2017) 2, no. 3, 10.3390/jfmk2030030.

[7] Desyibelew H. D. and Dadi A. F. , Burden and Determinants of Malnutrition Among Pregnant Women in Africa: A Systematic Review and Meta-Analysis, PloS one. (2019) 14, no. 9, e0221712, 10.1371/journal.pone.0221712, 31490956. 31490956 PMC6730925

[8] Bailey R. L. , Pac S. G. , Fulgoni V. L. , Reidy K. C. , and Catalano P. M. , Estimation of Total Usual Dietary Intakes of Pregnant Women in the United States, JAMA Network Open. (2019) 2, no. 6, e195967, 10.1001/jamanetworkopen.2019.5967, 31225890. 31225890 PMC6593963

[9] Bitew Z. W. , Alemu A. , Ayele E. G. , and Worku T. , Dietary Diversity and Practice of Pregnant and Lactating Women in Ethiopia: A Systematic Review and Meta-Analysis, Food Science & Nutrition. (2021) 9, no. 5, 2686–2702, 10.1002/fsn3.2228, 34026082. 34026082 PMC8116864

[10] Fite M. B. , Tura A. K. , Yadeta T. A. , Oljira L. , and Roba K. T. , Prevalence and Determinants of Dietary Practices Among Pregnant Women in Eastern Ethiopia, BMC Nutrients. (2022) 8, no. 1, 10.1186/s40795-021-00494-4, 35012664. PMC8751267 35012664

[11] Yalewdeg M. , Birhane M. , and Adissu Y. , Dietary Practices and Their Determinants Among Pregnant Women in Gedeo zone, Southern Ethiopia: A Community-Based Cross-Sectional Study, Nutrition and Dietary Supplements. (2020) 12, 267–275, 10.2147/NDS.S267453.

[12] Bayked E. M. , Yimer E. M. , Gelaw T. , Mohammed A. S. , and Mekonen N. A. , Dietary Knowledge, Attitude, Practice, and Associated Factors Among Pregnant Mothers in Ethiopia: A Systematic Review and Meta-Analysis, Frontiers in Public Health. (2024) 12, 1393764, 10.3389/fpubh.2024.1393764, 39328997. 39328997 PMC11425043

[13] Bayissa Z. B. , Girma T. N. , Alamirew J. A. , Fite R. O. , Alemu K. , Taddesse L. , Bekele D. , Tolera G. , Chan G. J. , Papatheodorou S. I. , and Gelaye B. , Dietary Diversity and Associated Factors Among Pregnant Women in Ethiopia: A Systematic Review With Meta-Analysis, Journal of Global Health. (2025) 15, 04286, 10.7189/jogh.15.04286, 41132055. 41132055 PMC12550538

[14] Tesfa S. , Alemu Z. , Tesfaye A. , Abebe H. , and Tsehay T. , Maternal Nutritional Knowledge, Practice and Their Associated Factors During Pregnancy in Addis Sub city Health Centers, Addis Ababa, Ethiopia, International Journal of Africa Nursing Sciences. (2022) 17, 100482, 10.1016/j.ijans.2022.100482.

[15] Geta T. G. , Gebremedhin S. , and Omigbodun A. O. , Dietary Diversity Among Pregnant Women in Gurage Zone, South Central Ethiopia: Assessment Based on Longitudinal Repeated Measurement, International Journal of Women′s Health. (2022) 14, 599–615, 10.2147/IJWH.S354536, 35497262. PMC9048948 35497262

[16] Alamirew S. K. , Lemke S. , Freyer B. , and Stadlmayr B. , Dietary Behaviour of Pregnant Women in Ethiopia: The Missing Aspect of Care, Nutrients. (2024) 16, no. 19, 10.3390/nu16193227, 39408195. PMC11478598 39408195

[17] Ethiopia F. D. R. O. , Mo H. , National Nutrition Program Multisectoral Implementation Guide, 2016, Addis Ababa.

[18] Tolera B. , Mideksa S. , and Dida N. , Assessment of Dietary Practice and Associated Factors Among Pregnant Mother in Ambo District, West Shoa, Oromia, Ethiopia, 2018, Ethiopian Journal of Reproductive Health. (2018) 10, no. 4, 10.69614/ejrh.v10i4.205.

[19] Kuche D. , Singh P. , and Moges D. , Dietary Practices and Associated Factors Among Pregnant Women IN Wondo Genet District, Southern Ethiopia: A Cross-Sectional Study, Journal of Pharmaceutical & Scientific Innovation. (2015) 4, no. 5, 270–275, 10.7897/2277-4572.04560.

[20] Kennedy G. , Ballard T. , and Dop M. , Food and Agriculture Organization Guidelines for Measuring Household and Individual Dietary Diversity, 2013, Nutrition and Consumer Protection Division, Food and Agriculture Organization of the United Nations.

[21] Girma A. , Genetu A. , Ayalew E. , and Getachew D. , Determinants of Dietary Practice Among Pregnant Women at the Public Hospitals in Bench-Sheko and Kaffa Zones, Southwest Ethiopia, Southwest Ethiopia. BMC Nutrition. (2022) 8, no. 1, 1–12, 10.1186/s40795-022-00588-7. PMC9400258 36002906

[22] Girma Tilahun A. , Molla Kebede A. , and Ejigu A. G. , Dietary Practice and Associated Factors Among Pregnant Women at Public Health Institution in Mizan-Aman Town, Southwest Ethiopia, Southwest Ethiopia. Nutrition and Metabolic Insights. (2021) 14, 11786388211057796, 10.1177/11786388211057796, 34819735. 34819735 PMC8606983

[23] Tsegaye D. , Tamiru D. , and Belachew T. , Factors Associated With Dietary Practice and Nutritional Status of Pregnant Women in Rural Communities of Illu aba Bor zone, Southwest Ethiopia, Nutrition and Dietary Supplements. (2020) 12, no. 103, 103–112, 10.2147/NDS.S257610.

[24] FAO , Minimum Dietary Diversity for Women, 2021, FAO, 10.4060/cb3434en.2021.

[25] Ballard T. , Coates J. , Swindale A. , and Deitchler M. , Household Hunger Scale: Indicator Definition and Measurement Guide, Food and Nutrition Technical Assistance Project II. (2011) 360, https://www.fao.org/fileadmin/user_upload/wa_workshop/docs/HH_Hunger_Scale.pdf.

[26] Ververs M. T. , Antierens A. , Sackl A. , Staderini N. , and Captier V. , Which Anthropometric Indicators Identify a Pregnant Woman as Acutely Malnourished and Predict Adverse Birth Outcomes in the Humanitarian Context?, Plos Currents. (2013) 5, 10.1371/currents.dis.54a8b618c1bc031ea140e3f2934599c8. PMC3682760 23787989

[27] Tang A. M. , Dong K. , Deitchler M. , Chung M. , Maalouf-Manasseh Z. , Tumilowicz A. et al., Use of Cutoffs for Mid-Upper Arm Circumference (MUAC) as an Indicator or Predictor of Nutritional and Health-Related Outcomes in Adolescents and Adults: A Systematic Review, 2013, FHI.

[28] Tang Y. Y. , Tang R. , and Posner M. I. , Brief Meditation Training Induces Smoking Reduction, Proceedings of the National Academy of Sciences. (2013) 110, no. 34, 13971–13975, 10.1073/pnas.1311887110, 23918376. PMC3752264 23918376

[29] Zewdie S. , Fage S. G. , Tura A. K. , and Weldegebreal F. , Undernutrition Among Pregnant Women in Rural Communities in Southern Ethiopia, Journal of Women′s Health. (2021) 13, 73–79, 10.2147/IJWH.S285132. PMC7802823 33447094

[30] Qureshi Z. and Khan R. , Dietary Intake Trends Among Pregnant Women in Rural Area of Rawalpindi, Pakistan, Journal of Ayub Medical College Abbottabad. (2015) 27, no. 3, 684–688. 26721040

[31] Olatona F. A. , Olowu O. J. , Goodman O. O. , and Amu E. O. , Dietary Habits, Diversity, and Predictors Among Pregnant Women Attending Primary Health Care Centers for Antenatal Care in Lagos, Nigeria, Journal of Family Medicine and Primary Care. (2021) 10, no. 8, 3076–3083, 10.4103/jfmpc.jfmpc_397_21, 34660450. PMC8483102 34660450

[32] Aliwo S. , Fentie M. , Awoke T. , and Gizaw Z. , Dietary Diversity Practice and Associated Factors Among Pregnant Women in North East Ethiopia, BMC Research Notes. (2019) 12, no. 1, 1–6, 10.1186/s13104-019-4159-6. 30845950 PMC6407270

[33] Hoffmann J. F. , Nunes M. A. A. , Schmidt M. I. , Olinto M. T. A. , Melere C. , Ozcariz S. G. I. , Buss C. , Drhemer M. , Manzolli P. , Soares R. M. , Pinheiro A. P. , and Camey S. , Dietary Patterns During Pregnancy and the Association With Sociodemographic Characteristics Among Women Attending General Practices in Southern Brazil: The ECCAGe Study, Cadernos de Saude Publica. (2013) 29, no. 5, 780–970, 10.1590/S0102-311X2013000500014, 23703002. 23703002

[34] Hailu S. and Woldemichael B. , Dietary Diversity and Associated Factors Among Pregnant Women Attending Antenatal Care at Public Health Facilities in Bale Zone, Southeast Ethiopia, Nutrition and Dietary Supplements. (2019) 11, 1–8, 10.2147/NDS.S179265.

[35] Wojtyła A. , Bojar I. , Boyle P. , Zatoński W. , Marcinkowski J. T. , and Biliński P. , Nutritional Behaviours Among Pregnant Women From Rural and Urban Environments in Poland, Annals of Agricultural and Environmental Medicine: AAEM. (2011) 18, no. 1, 169–174, 21736282. 21736282

[36] Azene A. G. , Aragaw A. M. , Wubetie H. T. , Wassie G. T. , Tsegaye G. W. , Derebe M. A. , and Mitiku H. D. , Dietary Diversity Among Pregnant Women and Associated Factors in Ethiopia: Systematic Review and Meta-Analysis, PLoS One. (2021) 16, no. 6, e0251906, 10.1371/journal.pone.0251906, 34111140. 34111140 PMC8191951

[37] Kibr G. , A Narrative Review of Nutritional Malpractices, Motivational Drivers, and Consequences in Pregnant Women: Evidence From Recent Literature and Program Implications in Ethiopia, Scientific World Journal. (2021) 2021, 5580039, 10.1155/2021/5580039, 34248425. 34248425 PMC8236338

[38] Abas A. H. , Ahmed A. T. , Farah A. E. , and Wedajo G. T. , Barriers to Optimal Maternal and Child Feeding Practices in Pastoralist Areas of Somali Region, Eastern Ethiopia: A Qualitative Study, Food and Nutrition Sciences. (2020) 11, no. 6, 540–561, 10.4236/fns.2020.116038.

[39] Nana A. and Zema T. , Dietary Practices and Associated Factors during pregnancy in Northwestern Ethiopia, BMC Pregnancy and Childbirth. (2018) 18, no. 1, 1–8, 10.1186/s12884-018-1822-1. 29801471 PMC5970492

[40] Belay W. S. , Cherkos E. A. , and Taye E. B. , Dietary Practice During Pregnancy and Associated Factors Among Pregnant Women in Farta District, South Gondar Zone, Northwest Ethiopia 2021, Clinical Epidemiology and Global Health. (2022) 14, 100968, 10.1016/j.cegh.2022.100968.

[41] Kang Y. , Hurley K. M. , Ruel-Bergeron J. , Monclus A. B. , Oemcke R. , Wu L. S. F. , Mitra M. , Phuka J. , Klemm R. , West K. P. , and Christian P. , Household Food Insecurity Is Associated With Low Dietary Diversity Among Pregnant and Lactating Women in Rural Malawi, Public Health Nutrition. (2019) 22, no. 4, 697–705, 10.1017/S1368980018002719, 30378520. 30378520 PMC10260502

[42] Na M. , Mehra S. , Christian P. , Ali H. , Shaikh S. , Shamim A. A. , Labrique A. B. , Klemm R. D. W. , Wu L. S. F. , and West K. P. , Maternal Dietary Diversity Decreases With Household Food Insecurity in Rural Bangladesh: A Longitudinal Analysis, Journal of Nutrition.(2016) 146, no. 10, 2109–2116, 10.3945/jn.116.234229, 27581578. 27581578

[43] Demilew Y. M. , Alene G. D. , and Belachew T. , Dietary Practices and Associated Factors Among Pregnant Women in West Gojjam Zone, Northwest Ethiopia, BMC Pregnancy and Childbirth. (2020) 20, no. 1, 1–11, 10.1186/s12884-019-2702-z. PMC6945405 31906981

